# An Observational Study to Evaluate Dry Eye Status in Type 2 Diabetes Mellitus Patients and Its Association With Diabetic Retinopathy

**DOI:** 10.7759/cureus.105279

**Published:** 2026-03-15

**Authors:** Rahul Prasad, Nitish Marshal, Komal Soni, Priya Suman, Ashish Bharti

**Affiliations:** 1 Ophthalmology, Regional Institute of Ophthalmology, Rajendra Institute of Medical Sciences, Ranchi, IND; 2 Ophthalmology, Rajendra Institute of Medical Sciences, Ranchi, IND

**Keywords:** diabetic retinopathy, dry eye disease, osdi, schirmer test, tbut, type 2 diabetes mellitus

## Abstract

Introduction

Type 2 diabetes mellitus affects millions globally and frequently leads to ocular complications. This study investigated the prevalence of dry eye disease in diabetic patients and examined whether tear film dysfunction correlates with diabetic retinopathy severity.

Methodology

We conducted a cross-sectional observational study at the Department of Ophthalmology, Regional Institute of Medical Sciences, Ranchi, from September 2023 to December 2024. We enrolled 153 consecutive patients with type 2 diabetes and assessed them using the Ocular Surface Disease Index questionnaire, tear break-up time measurement, Schirmer's test, and fluorescein staining. Diabetic retinopathy was graded using dilated indirect ophthalmoscopy.

Results

Among 153 diabetic patients (mean age 55.48 ± 8.66 years, 69.28% male), 64 (41.83%) had dry eye disease. Most cases were moderate severity (21.57%). Patients with dry eye had longer diabetes duration than those without (6.00 ± 2.88 vs 4.95 ± 2.03 years, t = 2.45, p = 0.01). Diabetic retinopathy was present in 48 cases (31.37%). Dry eye severity correlated significantly with retinopathy progression (χ² = 8.76, p < 0.05). Higher HbA1c levels are associated with increased dry eye prevalence (F = 12.34, p < 0.001), with mean HbA1c rising from 6.89 ± 0.49 in unaffected patients to 8.48 ± 1.24 in severe cases.

Conclusion

Dry eye disease is common among patients with type 2 diabetes. Advanced age, prolonged diabetes duration, and poor glycemic control are associated with higher dry eye prevalence. The strong correlation between diabetic retinopathy severity and dry eye suggests shared pathophysiological mechanisms. These findings support comprehensive ocular surface screening in diabetic patients alongside routine retinal examination.

## Introduction

Diabetes mellitus represents one of the most significant global health challenges of the 21st century, with projections indicating that 380 million individuals worldwide will be affected by 2025 [1]. The chronic hyperglycemic state characteristic of diabetes leads to numerous complications affecting both macrovascular and microvascular systems, resulting in diabetic neuropathy, nephropathy, and retinopathy. Among these complications, diabetic retinopathy (DR) stands as one of the most serious and common consequences, primarily caused by microvascular damage from chronic hyperglycemia [2].

While diabetic retinopathy has received considerable attention as a major diabetic complication, dry eye disease has emerged as an increasingly recognized ocular surface disorder in diabetic patients. Dry eye disease is a multifactorial condition characterized by inadequate lacrimal aqueous layer production and excessive tear evaporation, leading to ocular discomfort, visual disturbances, tear film instability, and potential ocular surface damage [3]. The prevalence of dry eye disease in diabetic patients varies considerably across studies, ranging from 18% to 70%, with hospital-based studies typically reporting higher rates than community-based investigations [4]. The pathophysiology of dry eye disease in diabetic patients involves multiple mechanisms, including peripheral neuropathy affecting corneal sensation, lacrimal gland dysfunction, inflammatory changes, and hyperglycemia-induced systemic hyperosmotic imbalances [5]. The International Dry Eye Workshop has identified that diminished corneal sensitivity contributes to dry eye syndrome through two primary pathways: disruption of lacrimal gland reflex circuits leading to reduced tear production and decreased blinking frequency resulting in increased tear evaporation [6].

Numerous studies have shown a strong link between dry eye disease and diabetic retinopathy, indicating common pathophysiological mechanisms. Manaviat et al. identified a significant link between dry eye disease, the duration of diabetes, and diabetic retinopathy, with as many as 54.3% of diabetics experiencing dry eye disease [2]. The Beaver Dam Eye Study found that around 20% of dry eye instances were present in people with type 2 diabetes mellitus aged 43 to 86 years [7]. The relationship between glycaemic management and dry eye disease has been thoroughly researched as well. Poor metabolic regulation, as shown by high HbA1c levels, has been linked to a higher occurrence and intensity of dry eye symptoms. Najafi et al. identified dry eye disease in 27.7% of individuals with diabetes and discovered a connection between dry eye disease and elevated HbA1c levels, implying that poor blood sugar management could play a role in this issue [8].

Tear hyperosmolarity is acknowledged as a significant contributor to the onset of ocular surface inflammation, tissue harm, and associated symptoms in dry eye disease. Decreased aqueous tear production and heightened tear evaporation can cause excessive fluid loss from the eye surface, leading to epithelial cell injury by activating inflammatory pathways and releasing inflammatory mediators into the tear film [9]. The incidence of diabetes is increasing swiftly, especially in developing countries such as India, due to the higher rates of obesity and poor lifestyle choices. Due to the elevated frequency of diabetic retinopathy and the growing awareness of dry eye as a notable complication, thorough research of ocular surface health in diabetic individuals has gained greater importance. The goal of this research is to explore the occurrence of dry eye disease among type 2 diabetes patients and to analyze its correlation with the severity of diabetic retinopathy, diabetes duration, and glycaemic control.

## Materials and methods

Study design

A cross-sectional observational study was performed within a clinical environment to determine the frequency of ocular surface disease among patients diagnosed with type 2 diabetes mellitus, while simultaneously examining potential associations between this condition and the presence of diabetic retinopathy.

Study population

This study examined patients with type 2 diabetes mellitus who received ophthalmological care at the Regional Institute of Ophthalmology, Rajendra Institute of Medical Sciences (RIMS), Ranchi, during the period from September 2023 through December 2024. The research population consisted of diabetic individuals seeking specialized eye care services at this tertiary care facility. Data collection spanned approximately fifteen months, encompassing patients who presented for routine diabetic retinopathy screening as well as those with existing ocular complications secondary to their metabolic condition.

Sampling strategy and potential bias

Participants were enrolled consecutively among type 2 diabetic patients presenting for ophthalmologic evaluation during the study period. Consecutive sampling was employed to ensure representative capture of the diabetic population seeking eye care at our tertiary center. We acknowledge that this approach may introduce selection bias, as patients attending our facility may have more advanced disease or higher comorbidity burden compared to the general diabetic population. Additionally, patients with existing ocular complaints may be overrepresented compared to community-based samples.

Confounder assessment

Data on systemic comorbidities, including hypertension and dyslipidemia, were collected through patient history and medical records. Duration of these comorbidities was documented when available. While these factors were recorded, the study design did not include matching or stratification for these variables, representing a limitation in confounder control that is addressed in our statistical approach and discussed in the limitations section.

Inclusion criteria

This study examined type 2 diabetic patients receiving routine eye care at our ophthalmology clinic. Participants included individuals with a documented type 2 diabetes diagnosis based on American Diabetes Association criteria: fasting plasma glucose ≥126 mg/dL (7.0 mmol/L), HbA1c ≥6.5%, two-hour plasma glucose ≥200 mg/dL (11.1 mmol/L) during an oral glucose tolerance test, or current use of anti-diabetic medications with a physician-confirmed type 2 diabetes diagnosis. All diagnoses were verified from medical records. Participants were recruited from consecutive diabetic individuals presenting for retinal screening during the enrollment period. Given diabetes-related eye complications, systematic evaluation of this patient population provides important insights into disease patterns and clinical outcomes.

Exclusion criteria

To minimize confounding variables, several exclusion criteria were established. Individuals taking medications affecting tear production, including hormonal contraceptives, tricyclic antidepressants, and antihistamines, were deemed ineligible. The study excluded contact lens users due to the potential impact of contact lenses on ocular surface moisture. Participants with prior ocular surgery, particularly laser-assisted in situ keratomileusis (LASIK) or intraocular procedures, were also excluded due to potential effects on tear film stability. Current tobacco users were not permitted to enroll due to smoking's known association with ocular surface dysfunction.

Additionally, participants who had used any topical ocular medications, including corticosteroids, lubricating eye drops, artificial tears, or other topical ophthalmic preparations within the preceding four weeks, were excluded from the study to avoid confounding effects on tear film assessment and dry eye measurements.

Sample size calculation

Based on the prevalence of dry eye syndrome reported as 42% in previous literature, using an 8% margin of error and 95% confidence interval, the estimated sample size was calculated as 153 participants [10]. The final study included 153 consecutive patients meeting the inclusion criteria.

Methodology

Following institutional ethics approval and obtaining written informed consent per Helsinki Declaration guidelines, we collected comprehensive data, including demographics (age, gender, occupation), diabetes-specific parameters (type, duration, treatment regimen, fasting glucose, HbA1c, and postprandial glucose), and detailed symptomatology. Dry eye assessment used the validated 12-item Ocular Surface Disease Index (OSDI) questionnaire [11], which covers visual function, ocular symptoms, and environmental triggers. Items were scored 0-4 points, with calculated OSDI values ((sum × 25) ÷ questions answered) categorizing severity as normal (0-12), mild (13-22), moderate (23-32), or severe (33-100).

Ophthalmic examination included Snellen visual acuity and slit-lamp biomicroscopy. Objective tear film evaluation employed: (1) fluorescein tear break-up time (TBUT) using 1% fluorescein strips, with <10 seconds indicating instability; (2) Schirmer testing using standard 5 × 35 mm Whatman strips at lateral canthus for five minutes, with <10 mm considered abnormal; and (3) ocular surface staining with fluorescein under cobalt blue illumination, graded using the Oxford scale (0-5) across five zones.

Posterior segment evaluation following mydriasis (tropicamide 0.8% and phenylephrine 5%) was performed using slit-lamp biomicroscopy with +90D non-contact lens and binocular indirect ophthalmoscopy with 20-diopter lens. Diabetic retinopathy was classified according to the International Clinical Diabetic Retinopathy Disease Severity Scale as mild, moderate, or severe non-proliferative diabetic retinopathy (NPDR), or proliferative diabetic retinopathy (PDR). While fundus photography and fluorescein angiography provide superior documentation and detailed characterization of retinal microvasculature, clinical examination remains an accepted method for diabetic retinopathy (DR) screening and staging in routine clinical practice, particularly in resource-limited settings [12]. All examinations were performed by experienced ophthalmologists (RP and NM) with demonstrated inter-observer reliability (Cohen's kappa = 0.85).

All ophthalmic measurements were performed by two trained ophthalmologists (RP and NM). Inter-observer agreement was assessed in a subset of 30 patients (19.6% of the sample) for TBUT, Schirmer testing, and DR grading. Cohen's kappa values demonstrated substantial agreement: TBUT (κ = 0.78), Schirmer (κ = 0.81), and DR grading (κ = 0.85), indicating good reliability of measurements.

Statistics

Data management and analysis were performed using the pandas library (version 1.5.3) in Python (version 3.10.8). Python is developed and maintained by the Python Software Foundation (PSF), a non-profit organization. The version used in this study was Python 3.10.8, released by the Python Software Foundation, Delaware, USA. Python was selected over traditional statistical software (SPSS, R, and Stata) for its robust data manipulation capabilities and reproducibility advantages. All analysis code was version-controlled and documented to ensure reproducibility; scripts are available from the corresponding author upon request. Descriptive statistics are presented as means ± standard deviations for continuous variables and frequencies with percentages for categorical variables. Comparative analyses employed independent-samples Student's t-tests for continuous variables between two groups, with results reported as t-statistic and p-value. One-way analysis of variance (ANOVA) with F-statistics assessed differences across multiple dry eye severity categories. Chi-square tests (χ²) examined associations between categorical variables, particularly dry eye presence and diabetic retinopathy stages. Ninety-five percent confidence intervals (95% CI) were calculated for all prevalence estimates and measures of association to provide precision estimates for our findings. All p-values are reported in standardized format with corresponding test statistics. Given the exploratory nature of multiple comparisons examining different aspects of dry eye associations, formal adjustment for multiple testing (e.g., Bonferroni correction) was not applied; p-values should be interpreted accordingly. Statistical significance was set at p ≤ 0.05 for all analyses.

While bivariate analyses effectively identified associations between dry eye disease and key diabetic parameters, formal multivariate logistic regression modeling to simultaneously control for multiple confounders (age, gender, diabetes duration, HbA1c, comorbidities) was limited by sample size considerations, particularly in stratified analyses by dry eye severity. This represents a limitation in our ability to establish independent risk factors and is addressed in the discussion.

## Results

Demographic characteristics

The study enrolled 153 consecutive patients with type 2 diabetes mellitus. Participants had a mean age of 55.48 ± 8.66 years (range: 42-81 years). Males predominated, comprising 106 participants (69.28%), with 47 females (30.72%). The mean diabetes duration was 6.00 ± 2.88 years. Dry eye disease was diagnosed in 64 participants (41.83%, 95% CI: 34.1%-49.9%). Among affected individuals, 19 (12.42%) had mild dry eye, 33 (21.57%) had moderate severity, and 12 (7.84%) presented with severe dry eye. The remaining 89 patients (58.17%) showed no evidence of dry eye disease (Table 1).

**Table 1:** Distribution of dry eye disease severity among type 2 diabetes mellitus patients.

Dry eye status	Frequency	Percentage
No dry eye	89	58.17
Mild dry eye	19	12.42
Moderate dry eye	33	21.57
Severe dry eye	12	7.84
Total	153	100

Association between demographics and dry eye disease

Age emerged as a significant factor in dry eye development. Patients with dry eye were significantly older than those without (57.59 ± 8.83 vs 52.99 ± 7.22 years; t = 3.42, p < 0.001, 95% CI of difference: 2.15-7.05 years). Dry eye prevalence increased markedly with age, affecting 71.05% of participants aged ≥60 years compared to 28.4% of those <60 years (χ² = 28.53, p < 0.001). Gender distribution showed comparable rates between males (42.45%, 45/106) and females (40.43%, 19/47), with no statistically significant difference (χ² = 0.05, p = 0.487, OR = 1.08, 95% CI: 0.55-2.14).

Diabetes duration showed a strong dose-response relationship with dry eye severity (F = 8.92, p < 0.001). Patients without dry eye had the shortest diabetes duration (4.96 ± 2.03 years), while those with severe dry eye had the longest duration (11.25 ± 3.67 years). Post-hoc analysis revealed that severe dry eye patients had significantly longer diabetes duration than all other groups (p < 0.01 for all comparisons). Glycemic control, measured by HbA1c, demonstrated a powerful association with dry eye severity (F = 15.67, p < 0.001). Mean HbA1c levels progressively increased across dry eye categories: no dry eye (6.89 ± 0.49%), mild (6.95 ± 0.79%), moderate (7.65 ± 1.30%), and severe (8.48 ± 1.24%). The difference in HbA1c between patients without dry eye and those with severe dry eye was 1.59% (95% CI: 1.02-2.16%, p < 0.001) (Table 2).

**Table 2:** Association of dry eye disease severity with diabetes duration and glycemic control.

Dry eye status	Mean diabetes duration (years) ± SD	Mean HbA1c ± SD	Frequency
No dry eye	4.96 ± 2.03	6.89 ± 0.49	89
Mild dry eye	5.26 ± 2.13	6.95 ± 0.79	19
Moderate dry eye	7.48 ± 2.27	7.65 ± 1.30	33
Severe dry eye	11.25 ± 3.67	8.48 ± 1.24	12
Total	6.03 ± 2.89	7.19 ± 0.96	153

Diabetic retinopathy prevalence and distribution

Diabetic retinopathy was present in 48 patients (31.37%, 95% CI: 24.2%-39.3%). Disease severity distribution included: mild non-proliferative diabetic retinopathy (NPDR) in 3 patients (1.96%), moderate NPDR in 12 patients (7.84%), severe NPDR in 18 patients (11.76%), and proliferative diabetic retinopathy (PDR) in 15 patients (9.80%). No cases of very severe NPDR were observed.

Association between dry eye disease and diabetic retinopathy

A significant association existed between dry eye presence and diabetic retinopathy (χ² = 12.34, p = 0.006). Among patients with dry eye disease, 45.31% (29/64) had concurrent diabetic retinopathy compared to 21.35% (19/89) without dry eye (OR = 3.05, 95% CI: 1.52-6.13, p < 0.001). The relationship intensified with increasing dry eye severity (χ² for trend = 18.76, p < 0.001). Diabetic retinopathy prevalence was: 26.31% (5/19) in mild dry eye, 48.48% (16/33) in moderate dry eye, and 66.67% (8/12) in severe dry eye cases. Conversely, among patients with diabetic retinopathy, dry eye prevalence increased with retinopathy severity. Among those without retinopathy, 33.33% (35/105) had dry eye. This proportion rose to 66.67% (2/3) in mild NPDR, decreased to 8.33% (1/12) in moderate NPDR, increased to 72.22% (13/18) in severe NPDR, and reached 86.67% (13/15) in proliferative diabetic retinopathy (Table 3).

**Table 3:** Association between dry eye disease severity and diabetic retinopathy stages. DR: diabetic retinopathy; NPDR: non-proliferative diabetic retinopathy; PDR: proliferative diabetic retinopathy.

Dry eye status	No DR	Mild NPDR	Moderate NPDR	Severe NPDR	PDR	Total
No dry eye	70 (66.67%)	1 (33.33%)	11 (91.67%)	5 (27.78%)	2 (13.33%)	89 (58.17%)
Mild dry eye	14 (13.33%)	1 (33.33%)	1 (8.33%)	1 (5.56%)	2 (13.33%)	19 (12.42%)
Moderate dry eye	17 (16.19%)	1 (33.33%)	0	10 (55.56%)	5 (33.33%)	33 (21.57%)
Severe dry eye	4 (3.81%)	0	0	2 (11.11%)	6 (40.00%)	12 (7.84%)
Total	105 (100%)	3 (100%)	12 (100%)	18 (100%)	15 (100%)	153 (100%)

Diagnostic test performance

Multiple diagnostic modalities demonstrated concordant patterns. OSDI questionnaire identified symptomatic dry eye in 40.52% of patients (62/153), with 26.80% reporting mild symptoms, 5.23% moderate, and 8.50% severe. Schirmer testing was abnormal in 32.68% (50/153): 7.84% mild, 16.34% moderate, and 8.50% severe reduction. TBUT identified instability in 39.87% (61/153): 32.03% with mild-moderate and 7.84% with severe reduction. Ocular surface staining revealed abnormalities in 35.95% (55/153): 5.88% mild, 21.57% moderate, and 8.50% severe staining.

## Discussion

The association between type 2 diabetes mellitus and ocular surface disorders has gained considerable attention in recent clinical investigations. Our research reveals that approximately two-fifths of diabetic individuals examined exhibited signs of dry eye syndrome, corroborating findings from comparable institutional research conducted by multiple investigators [10,13,14]. These hospital-based investigations consistently demonstrate elevated incidence rates compared to population-based epidemiological surveys, which typically report substantially lower frequencies of ocular surface disease in diabetic cohorts. The enhanced prevalence observed in clinical settings likely reflects the concentration of patients with more advanced glycaemic dysregulation and metabolic complications, suggesting that the severity of diabetes management may directly influence the development of associated ocular surface pathology. These findings underscore the importance of routine ophthalmological screening in diabetic patients receiving specialized medical care.

While the association between diabetes mellitus and dry eye disease has been established in previous literature from various geographic regions, significant variations exist in reported prevalence rates (18-70%) across different populations [4]. Regional differences in genetic susceptibility, environmental factors, dietary patterns, and healthcare access necessitate population-specific data to inform local clinical practice and public health strategies. This study contributes evidence from Eastern India (Jharkhand), a region with limited published data on diabetic ocular complications. Beyond prevalence estimation, our findings demonstrate important dose-response relationships between glycemic control (HbA1c levels) and dry eye severity, with mean HbA1c rising progressively from 6.89% in unaffected patients to 8.48% in severe cases. This graded relationship suggests potential HbA1c thresholds that could inform risk stratification for dry eye screening in diabetic populations.

Our findings demonstrate a clear correlation between advancing age and the development of dry eye syndrome within the diabetic population studied. Participants diagnosed with dry eye syndrome exhibited a statistically significant elevation in mean age compared to their unaffected counterparts, with affected individuals averaging 57.59 ± 8.83 years versus 52.99 ± 7.22 years in the control group. These results align with contemporary research frameworks, including the work of Cousen et al., which established age as a primary determinant in dry eye pathogenesis [13]. This age-related pattern is further supported by epidemiological studies, such as the work by Kaiserman et al., who documented increasing dry eye incidence with advancing age in older populations [15]. The striking observation that nearly three-quarters of participants beyond their sixth decade (71.05%) presented with dry eye symptoms reinforces the clinical significance of chronological age in predicting ocular surface disease risk among diabetic cohorts. This age-dependent pattern suggests that targeted screening protocols should prioritize older diabetic patients to facilitate early detection and intervention strategies.

Gender analysis revealed no statistically significant difference in dry eye disease prevalence between males (42.45%) and females (40.43%), which corroborates findings from several previous studies. Manaviat et al. reported similar non-significant gender associations, with 58% prevalence in females and 48.8% in males [2]. This finding contrasts with some general population studies that show female predominance in dry eye disease, suggesting that diabetes may override typical gender-related risk factors for dry eye development.

The relationship between diabetes duration and dry eye disease severity demonstrated in this study provides crucial clinical insights. The progressive increase in dry eye severity with longer diabetes duration, from 4.96 years in patients without dry eye to 11.25 years in those with severe dry eye, highlights the chronic progressive nature of diabetic complications affecting the ocular surface. This finding is consistent with multiple previous studies, including those by Manaviat et al. and Moss et al., which found strong correlations between diabetes duration and dry eye severity [2,16]. Similarly, Nepp et al. demonstrated comparable associations between diabetes duration, glycemic control, and dry eye prevalence in their study of type 2 diabetic patients [17].

The correlation between HbA1c levels and dry eye disease severity represents one of the most significant findings of this study. The progressive increase in HbA1c levels from 6.89 in patients without dry eye to 8.48 in those with severe dry eye demonstrates the critical role of glycaemic control in ocular surface health. This finding aligns with research by Najafi et al., who observed a relationship between dry eye disease and elevated HbA1c levels, suggesting that poor blood sugar control contributes to this condition [8]. The mechanism underlying this relationship likely involves chronic hyperglycemia leading to systemic inflammation, osmotic imbalances, and direct effects on lacrimal gland function.

Our investigation demonstrates a compelling correlation between diabetic retinopathy severity and dry eye syndrome occurrence, suggesting shared pathogenic mechanisms in diabetic ocular complications. The progressive increase in dry eye prevalence across retinopathy stages, reaching 86.67% in proliferative cases, indicates overlapping disease pathways. These findings support previous work by Stapleton et al., who observed escalating dry eye severity with advancing retinopathy [18]. The robust association suggests that common inflammatory or microvascular processes may simultaneously affect retinal integrity and tear film stability, highlighting the need for comprehensive ocular evaluation in diabetic patients.

Several mechanisms may explain the observed association between diabetic retinopathy and dry eye disease. Both conditions share common pathophysiological factors, including oxidative stress, inflammation, and diabetic neuropathy. The progressive damage to corneal nerve fibers in diabetic neuropathy impairs corneal sensation, which is critical for triggering reflex tear production. As diabetic retinopathy advances, representing more severe and prolonged hyperglycemic damage, the corresponding deterioration in ocular surface health and tear film stability becomes more pronounced.

The diagnostic utility of various dry eye assessment tools demonstrated in this study provides practical clinical guidance. The OSDI questionnaire identified symptomatic dry eye in 40.52% of patients, closely matching the overall prevalence detected through comprehensive evaluation. The Schirmer test showed somewhat lower sensitivity at 32.68%, while TBUT and ocular surface staining provided intermediate detection rates. This variability emphasizes the importance of comprehensive multi-modal evaluation for accurate dry eye diagnosis in diabetic patients, as recommended by international dry eye guidelines [19].

The moderate dry eye category was the most prevalent (21.57%) in this study, which differs from some previous reports that found mild dry eye as the most common. This finding may reflect the hospital-based nature of the study population, which likely included patients with more advanced or poorly controlled diabetes compared to community-based populations. The relatively high proportion of moderate-to-severe dry eye cases underscores the clinical significance of this complication in diabetic patients.

These results have important clinical relevance. The significant associations observed between dry eye disease and factors such as diabetes duration, glycemic control (HbA1c), and diabetic retinopathy severity suggest that ocular surface assessment should be considered as part of comprehensive eye care for diabetic patients. However, our cross-sectional study design precludes establishing causal relationships; these associations require validation through prospective longitudinal studies before definitive screening recommendations can be made. Spotting and treating dry eye early could boost patients' quality of life and help avoid more severe eye surface issues.

Study limitations

This investigation has several limitations that warrant consideration. The cross-sectional design precludes establishing temporal or causal relationships between dry eye disease and diabetic retinopathy; longitudinal studies are needed to determine whether dry eye precedes, follows, or develops concurrently with retinal complications. Consecutive sampling from a single tertiary care center may introduce selection bias, as our population likely represents patients with more advanced or symptomatic disease compared to community-dwelling diabetics. This hospital-based sample may therefore overestimate true population prevalence and limit generalizability.

Sample size considerations also warrant discussion. While our calculated sample size (n=153) was adequate for estimating overall dry eye prevalence with acceptable precision (95% CI: 34.1%-49.9%), it limited our statistical power for detecting associations within smaller subgroups, particularly individual diabetic retinopathy stages. The consecutive sampling approach, though ensuring representative capture of patients seeking tertiary eye care during the study period, introduced inherent selection bias. Patients attending our specialized ophthalmology department likely represent a population with more advanced diabetic complications or higher symptom burden compared to community-dwelling diabetics. This hospital-based sampling frame may overestimate true population prevalence and limit the generalizability of our findings to the broader type 2 diabetic population. Future multi-center studies with random community-based sampling would provide more generalizable prevalence estimates.

Our study did not employ matching or stratification for important confounders such as duration of hypertension and dyslipidemia, and the relatively modest sample size precluded comprehensive multivariate modeling to simultaneously adjust for multiple covariates. Future studies with larger samples should employ logistic regression to identify independent risk factors. While we assessed inter-observer reliability in a subset of patients with satisfactory results, formal evaluation of intra-observer variability was not performed.

The use of clinical examination for diabetic retinopathy grading without fundus photography or fluorescein angiography represents a limitation. While binocular indirect ophthalmoscopy is an established clinical method with demonstrated reliability in our study (κ=0.85), fundus photography would have provided objective documentation and potentially more precise grading, particularly for subtle early changes. Future studies should incorporate imaging modalities for enhanced accuracy and documentation.

Our study assessed dry eye disease globally using validated clinical measures (OSDI, TBUT, Schirmer test, and fluorescein staining) without differentiating between aqueous-deficient and evaporative dry eye subtypes. We did not perform meibography or specifically evaluate meibomian gland dysfunction (MGD), which represents an important etiological component of dry eye pathophysiology. While the Schirmer test assesses aqueous production and TBUT indicates tear stability, comprehensive subtype classification requires additional specialized testing, including meibomian gland imaging, interferometry for lipid layer assessment, and tear film osmolarity measurement. The relative contributions of aqueous deficiency versus evaporative dysfunction to dry eye disease in diabetes remain incompletely characterized. Future studies should incorporate meibomian gland assessment, tear osmolarity measurement, and lipid layer evaluation to clarify which dry eye subtypes show the strongest associations with diabetes and diabetic retinopathy.

Advanced diagnostic techniques not employed in this study include tear osmolarity measurement (considered the gold standard for dry eye diagnosis), interferometry for non-invasive tear film lipid layer assessment, meibography for meibomian gland structural imaging, in vivo confocal microscopy for corneal nerve density quantification, and conjunctival impression cytology for ocular surface cellular characterization. These technologies would provide more comprehensive insights into tear film composition, meibomian gland morphology, corneal nerve integrity, and inflammatory markers on the ocular surface. Future investigations incorporating these advanced modalities would offer superior characterization of dry eye pathophysiology in diabetes and potentially identify specific biomarkers correlating with disease progression. However, our multi-modal clinical assessment approach using validated, internationally standardized tests (OSDI, TBUT, Schirmer, and fluorescein staining) provides clinically relevant data applicable to routine practice settings globally, including resource-limited environments where advanced equipment may not be accessible.

Finally, our analysis relied on Python/pandas rather than traditional clinical research platforms; however, version control and code documentation ensure reproducibility of our findings.

## Conclusions

In this hospital-based cohort of type 2 diabetic patients, dry eye disease prevalence reached approximately 42%, with significant associations observed between dry eye severity and key diabetic parameters, including age, diabetes duration, and glycemic control (HbA1c). The observed correlation between dry eye syndrome and diabetic retinopathy progression, particularly in advanced proliferative stages, suggests potentially shared pathophysiological mechanisms involving microvascular damage, neuropathy, and chronic inflammation. While these findings are limited by the single-center design, consecutive hospital-based sampling, and cross-sectional methodology that precludes causal inference, the demonstrated dose-response relationships between HbA1c levels and dry eye severity warrant further investigation in larger, multi-center prospective studies. The mean HbA1c difference of 1.59% between unaffected patients and those with severe dry eye suggests potential thresholds for risk stratification.

Integration of dry eye screening into routine ophthalmologic assessments for diabetic patients may represent an important preventive strategy, though prospective studies are needed to establish clinical benefit and cost-effectiveness before definitive screening recommendations can be formulated. Future research should employ fundus photography for objective diabetic retinopathy documentation, incorporate advanced dry eye diagnostic modalities (tear osmolarity, meibography, interferometry), differentiate between dry eye subtypes, and utilize multivariate modeling to identify independent risk factors. Longitudinal studies are particularly needed to establish temporal relationships and determine whether dry eye progression parallels or predicts diabetic retinopathy advancement, which could inform personalized screening intervals and therapeutic interventions for this vulnerable population.
